# Low quality of life in men with chronic prostatitis-like symptoms

**DOI:** 10.1038/s41391-022-00559-w

**Published:** 2022-06-25

**Authors:** Mikio Sugimoto, Yasukazu Hijikata, Yoichiro Tohi, Hideya Kuroda, Mineo Takei, Takakazu Matsuki, Tsukasa Kamitani, Yoshiyuki Kakehi, Yosuke Yamamoto, Shunichi Fukuhara

**Affiliations:** 1 Department of Urology, Faculty of Medicine, Kagawa University, Kagawa, Japan; 2 Department of Healthcare Epidemiology, School of Public Health in the Graduate School of Medicine, Kyoto University, Kyoto, Japan; 3 Section of Clinical Epidemiology, Department of Community Medicine, Graduate School of Medicine, Kyoto University, Kyoto, Japan; 4 Kuroda Urology Clinic, Osaka, Japan; 5 Department of Urology, Harasanshin General Hospital, Fukuoka, Japan; 6 Matsuki Urology Clinic, Kagawa, Japan; 7 Section of Education for Clinical Research, Kyoto University Hospital, Kyoto, Japan; 8 Department of Health Policy and Management, Johns Hopkins Bloomberg School of Public Health, Baltimore, USA; 9 Shirakawa STAR for General Medicine, Fukushima Medical University, Fukushima, Japan

**Keywords:** Prostatitis, Outcomes research

## Abstract

**Background:**

Chronic prostatitis (CP) can impair health-related quality of life (QOL), but the full impact of CP, including the impact of CP-like symptoms in men who have no CP diagnosis (CPS), is unknown. We estimated the impact of diagnosed CP (DCP) and CPS on Health-related QOL.

**Methods:**

From a representative nationwide survey of men aged 20–84 in Japan, we determined the prevalence of DCP and also of CPS. For CPS, we used Nickel’s criteria, which were used previously to estimate the prevalence of CP and are based on the NIH Chronic Prostatitis Symptom Index. To test the robustness of Nickel’s criteria, we used two other definitions of CPS (two sensitivity analyses). We measured QOL with the Short-Form 12-Item Health Survey. We compared the participants’ QOL scores with the national-norm scores, and with the scores of men who had benign prostatic hyperplasia (BPH).

**Results:**

Among the 5 010 participants, 1.4% had DCP and 3.7% had CPS. The sensitivity analyses resulted in CPS prevalence estimates of 3.1% and 4.5%. CPS was particularly common in younger participants (5.7% of those in their 30 s had CPS). QOL was very low among men with CP: In most areas (domains) of QOL, their scores were more than 0.5 standard deviation below the national-norm mean. Their mental-health scores were lower than those of men with BPH. The lowest scores among all 8 QOL domains were in role-functioning.

**Conclusions:**

CP is common, but it is underdiagnosed, particularly in younger men. Whether diagnosed or only suspected, CP’s impact on QOL is large. Because CP is common, and because it substantially impairs individuals’ QOL and can also reduce societal productivity, it requires more attention. Specifically, needed now is a simple tool for urologists and for primary care providers, to identify men, particularly young men, whose QOL is impaired by CP.

## Introduction

Prostatitis is a common cause of visits to primary-care and urology clinics. Its lifetime prevalence is about 9% [1]. The International Prostatitis Collaborative Network, established by the National Institutes of Health (NIH), developed a four-category classification of prostatitis syndromes that is widely accepted [2]. Chronic prostatitis/chronic pelvic pain syndrome (CP), classified as category III, accounts for 90% of symptomatic prostatitis and has a complex and heterogeneous etiology [3]. CP can be extremely difficult to diagnose because signs of the condition are few, and because patients with CP can present with one or more of many different symptoms: pelvic pain, discomfort, dysuria, etc. The resulting difficulties in diagnosis have slowed the growth in knowledge about CP and there is concern that many men with CP have not received appropriate medical attention.

To structure the assessment of CP patients, the NIH-Chronic Prostatitis Symptom Index (NIH-CPSI) was developed in 1999 [4]. Although the NIH-CPSI is a scale developed to observe symptom trends in clinical studies, Nickel et al. attempted to use its pain subscale in an epidemiological study to estimate the prevalence of CP-like symptoms [5]. Subsequently, use of Nickel’s criteria has resulted in estimates of the lifetime prevalence of CP from 1.8% to 9.7%, depending on the study population and methodology [5–10]. Moreover, Roberts et al. found that 1.7% of respondents reported having been treated for CP in the past two years [11]. However, given the difficulties in diagnosing CP, its presence may often be suspected without being diagnosed, but knowledge of the prevalence of CP-like symptoms in men who have no CP diagnosis (CPS) is scarce. Furthermore, health-related quality of life (QOL) is low among men in whom CP has been diagnosed [12–15], and the QOL of men with CP-like symptoms has also received some attention [16, 17]. However, we are aware of no previous work focusing on men who had not received a diagnosis of CP but who nonetheless had CP-like symptoms, and in whom QOL was measured in a way that allows comparison with people who have other medical conditions and comparison with the general population.

We used a nationally representative sample of the adult population of Japan to estimate the prevalence of diagnosed CP (DCP) and of CPS, to study the differences between DCP and CPS, and to investigate QOL in DCP and in CPS.

## Materials/Subjects and methods

### Study design and population

This nationwide survey was part of the Norm Study 2021. The Norm Study is carried out every few years using quota sampling to investigate QOL and factors that may be associated with it. The previous such survey was conducted in 2016 [18, 19]. The participants were men aged 20 through 84 years at the time of the survey. Data from those who answered all the questions were included. Those who could not read or write Japanese were not included. An independent research company, the Nippon Research Center, distributed the questionnaire via the Internet from January 5 through March 4, 2021. The sample was drawn from a panel comprising about 1.4 million residents of all areas of Japan. The sample was designed to represent the general population of Japan at the time of the national census in 2015 [20], and individuals were included until the goal of 5000 men was reached. Japan was divided into five areas, and quotas were set by area and by age. This study was done in accordance with the World Medical Association’s Declaration of Helsinki and was approved by the institutional review boards of Kagawa University’s Faculty of Medicine and of the Institute for Health Outcomes and Process Evaluation Research. All participants provided written informed consent electronically.

### Data collection

#### Demographic characteristics

The survey was used to collect information about demographic characteristics, QOL, and prostatitis-like symptoms experienced during the previous year. We collected data on age, body-mass index, comorbidities, depressive symptoms, tobacco smoking, alcohol consumption, history of sexually transmitted diseases, male infertility, high prostate specific antigen (PSA) level, sexual activity, living status (living alone or not), full-time employment, annual household income, and formal schooling. The presence of depressive symptoms was identified using a 5-item Mental Health Inventory (cut-off score: ≤52) [21]. Sexual activity was defined as the number of sexual acts per month, including masturbation. A high PSA level was assumed if there was a response of ‘Yes’ to the question, ‘Have you ever been diagnosed with a high PSA level?’. Household income was defined as “high” if the annual income was 5 million yen or more (approximately US$48,000). The level of formal schooling was defined as “high” if the respondent reported having a university degree or higher.

#### Definitions of CP

First, we assigned participants who answered ‘yes’ to the question ‘Have you ever been diagnosed with CP?’ to the DCP group. We defined the CPS group as comprising those of the remaining participants who met Nickel’s criteria: perineal and/or ejaculatory pain/discomfort and a total NIH-CPSI pain subscale score (range 0–21) of 4 or greater [5]. We assigned all others to the “no CP” group. To test the sensitivity of the estimates of CPS prevalence to the specific content of Nickel’s criteria, we carried out two sensitivity analyses. For each of those two sensitivity analyses, we used a different definition of CPS. For the first sensitivity analysis we defined CPS as a score of 8 points or greater on the pain subscale of the NIH-CPSI [22]. For the second sensitivity analysis we defined CPS as a total score of 20 or greater on the NIH-CPSI, because that was the median score of CP patients in a previous study [23]. Then we excluded from those two CPS groups all participants who had none of the 12 typical symptoms of CP established by a panel of experts (Appendix). Thus, there were three definitions of CPS: Nickel’s criteria, the definition in the first sensitivity analysis, and the definition in the second sensitivity analysis. This is also described in Table 2.

#### QOL assessment

QOL was assessed with the Short-Form 12-Item Health Survey (SF-12) [24], a generic instrument for measuring QOL in the following domains: physical functioning (PF), role physical (RP), bodily pain (BP), general health (GH), vitality (VT), social functioning (SF), role emotional (RE), and mental health (MH). Higher scores indicate better QOL [25]. For the Norm Study, the scores on each domain were standardized to the general population of Japan. Specifically, the mean score in the general population was set to 50, and the standard deviation of scores in the general population was set to 10 [25]. We investigated QOL in participants with DCP, in those with CPS, and also in those with benign prostatic hyperplasia (BPH). The participants who were considered to have BPH were those who responded ‘yes’ to the question, ‘Have you ever been diagnosed with BPH?’.

### Statistical analyses

We first described the characteristics of all participants, and then compared the characteristics of the three groups: DCP, CPS, and no-CP. Continuous variables were summarized by median and interquartile range, and dichotomous variables by number and percentage. *P* values for differences in characteristics were calculated from the Kruskal–Wallis test for continuous variables and the χ^2^ test for dichotomous variables. Second, we described the prevalence of CPS according to the main definition (Nickel’s criteria), and according to the two definitions in the sensitivity analyses. Third, we described the prevalence of DCP and of CPS by age group. Finally, we analyzed the SF-12 scores for participants with DCP, for those with CPS, and for those with BPH. In addition, for participants in the DCP and CPS groups, we computed QOL scores of those with NIH-CPSI pain subscale scores of 4–7 and for those with scores of 8 or higher.

Data were analyzed using Stata version 15.1 (StataCorp LLC, College Station, TX, USA).

## Results

The survey request was distributed to 19,450 men, and 6671 (34.3%) indicated a willingness to participate. Of these, 5010 (75.1%) provided complete responses and their data were analyzed (Table 1). The prevalence of DCP was 1.4% and the prevalence of CPS was 3.7%. From the sensitivity analyses, the prevalence of CPS was 4.5% when “8 points or greater on the pain subscale of the NIH-CPSI” was used, and it was 3.1% when “20 points or greater on the total NIH-CPSI score” was used (Table 2).

**Table 1:** Background characteristics.

	All participants	DCP	CPS	No CP	*p* value
	5010	71 (1.4)	187 (3.7)	4752	
Age	51 (38–65)	55 (39–71)	46 (35–61)	51 (38–65)	0.009
Body mass index	23 (21–25)	24 (22–26)	23 (21–26)	23 (21–25)	0.24
Comorbidity					
Hypertension	1321 (26)	29 (41)	50 (27)	1242 (26)	0.02
Diabetes	560 (11)	24 (34)	25 (13)	511 (11)	<0.001
Heart disease	197 (3.9)	19 (27)	11 (5.9)	167 (3.5)	<0.001
Stroke	131 (2.6)	9 (13)	9 (4.8)	113 (2.4)	<0.001
Depression	294 (5.9)	12 (17)	28 (15)	254 (5.3)	<0.001
Depressive symptoms	1013 (20)	27 (38)	74 (40)	912 (19)	<0.001
Sexually transmitted diseases	147 (2.9)	8 (11)	22 (12)	117 (2.5)	<0.001
Male infertility	31 (0.6)	5 (7.0)	3 (1.6)	23 (0.5)	<0.001
High PSA level	154 (3.1)	12 (17)	14 (7.5)	128 (2.7)	<0.001
Lifestyle					
Smoking habit	1246 (25)	23 (32)	56 (30)	1167 (25)	0.08
Weekly drinking habit	2757 (55)	42 (59)	97 (52)	2618 (55)	0.54
Sexual activity (per month)	3 (0–10)	2 (1–5)	5 (2–12)	3 (0–10)	<0.001
Socioeconomic factors					
Solitude	968 (19)	16 (23)	35 (19)	917 (19)	0.77
Full-time employment	3037 (61)	37 (52)	108 (58)	2892 (61)	0.23
High household income	2459 (49)	34 (48)	88 (47)	2337 (49)	0.83
High education	3512 (70)	58 (82)	130 (70)	3324 (70)	0.1

Data are presented as number (%) or median (interquartile range). For continuous variables the *p* values are from the Kruskal–Wallis test, and for dichotomous variables they are from the χ2 test.

*CP* chronic prostatitis/chronic pelvic pain syndrome, *DCP* diagnosed CP, *CPS* CP-like symptoms in men who have no CP diagnosis, *PSA* prostate specific antigen.

**Table 2:** Prevalence for each CP definition.

	All participants 5010	Prevalence (95% CI)
DCP	71	1.4 (1.1–1.8)
CPS 1 (Nickel’s criteria): Perineal and/or ejaculatory pain/discomfort, and a total NIH-CPSI pain subscale score of 4 or greater, with no history of CP diagnosis	187	3.7 (3.2–4.3)
CPS 2 (first sensitivity analysis): “Yes” on any of the 12 rule-in questions, and a NIH-CPSI pain subscale score of 8 or greater, with no history of CP diagnosis	224	4.5 (3.9–5.1)
CPS 3 (second sensitivity analysis): “Yes” on any of the 12 rule-in questions, and a total NIH-CPSI score of 20 or greater, with no history of CP diagnosis	155	3.1 (2.6–3.6)

Data are presented as number (%).

*CI* confidence interval, *CP* chronic prostatitis/chronic pelvic pain syndrome, *DCP* diagnosed CP, *CPS* CP-like symptoms in men who have no CP diagnosis, *NIH-CPSI* the National Institutes of Health Chronic Prostatitis Symptom Index.

**Fig. 1:**
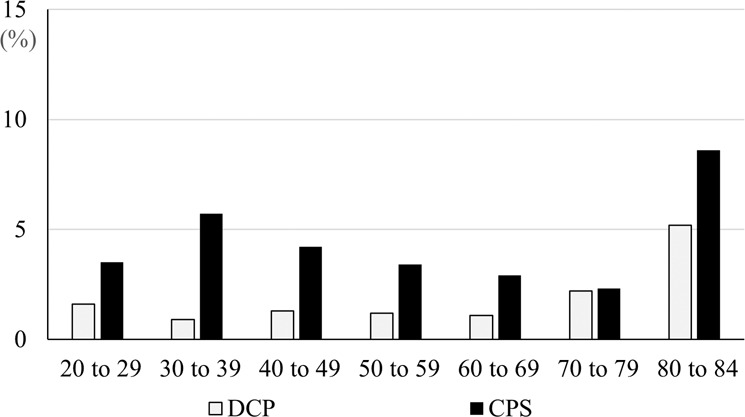
Age distributions of participants with diagnosed CP, and in those who had CP-like symptoms but who had no CP diagnosis (CPS). The vertical axis shows the prevalence (%) and the horizontal axis shows the age group. *CP* chronic prostatitis/chronic pelvic pain syndrome, DCP diagnosed CP, CPS CP-like symptoms in men who had no CP diagnosis.

The prevalence of DCP and of CPS differed greatly by age (Fig. 1). DCP was more prevalent among older participants, and it was most prevalent among those who were 80–84 years old. In contrast, the age distribution of CPS appeared to be bimodal, with peaks in the 30–39-year-old group and also in the 80–84-year-old group. The greatest difference in prevalence between DCP and CPS was in the 30–39-year-old group (0.9% for DCP vs 5.7% for CPS).

### Description of QOL (Table 3)

**Fig. 2:**
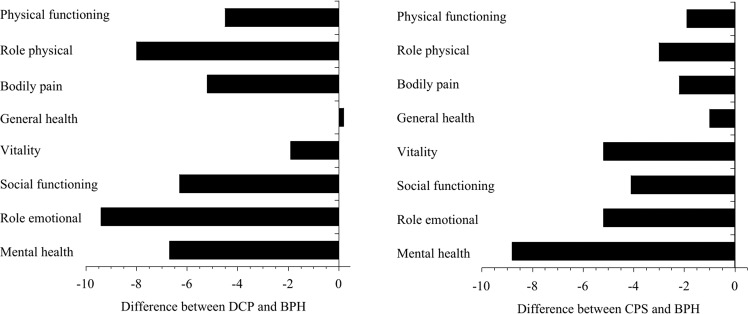
Differences in SF-12 scores between each CP definition and BPH, by domain. The length of each horizontal line denotes the difference in mean scores between those with DCP and those with BPH (left figure). The length of each horizontal line denotes the difference in mean scores between those with CPS and those with BPH (right figure). Values to the left of zero indicate that the QOL of participants with each CP definition was worse than the QOL of participants with BPH. CP chronic prostatitis/chronic pelvic pain syndrome, SF-12 Short-Form 12-Item Health Survey, DCP diagnosed CP, BPH benign prostatic hyperplasia, QOL quality of life, CPS CP-like symptoms in men who had no CP diagnosis.

The mean SF-12 scores of the participants with DCP, as well as the scores of those with CPS, were more than two points below the standardized national-norm mean value of 50 on all domains. In the participants with DCP, the scores on all domains except GH and VT were more than 0.5 standard deviation below the national-norm mean, and they were considerably lower than the scores of those who had BPH (Fig. 2A). The participants with DCP had scores on RP and RE that were more than one standard deviation below the national-norm mean. The participants with CPS had scores on RP, VT, SF, RE, and MH that were more than 0.5 standard deviation below the national-norm mean, all of which were considerably lower than those of participants with BPH (Fig. 2B). Those with NIH-CPSI pain subscale scores of 8 or higher had very low QOL. That low QOL was found not only in the DCP group but also in the CPS group (Supplementary Table 1).

**Table 3:** Scores on the domains of the SF-12.

	All participants	DCP	CPS	BPH
	*n* = 5010	*n* = 71	*n* = 187	*n* = 362
SF-12 domain				
Physical functioning	51.1 [10.4]	44.0 [15.1]	46.6 [13.8]	48.5 [12.7]
Role physical	49.2 [11.1]	38.1 [15.4]	43.1 [14.6]	46.1 [13.3]
Bodily pain	49.9 [10.6]	42.2 [13.1]	45.2 [10.9]	47.4 [11.7]
General health	50.8 [10.9]	47.2 [12.1]	46.0 [12.4]	47.0 [11.3]
Vitality	46.5 [10.5]	46.9 [9.2]	43.6 [9.5]	48.8 [10.0]
Social functioning	49.4 [11.4]	42.2 [13.4]	44.4 [14.1]	48.5 [12.6]
Role emotional	49.4 [10.9]	38.7 [14.0]	42.9 [13.0]	48.1 [12.4]
Mental health	48.3 [10.5]	44.7 [10.0]	42.6 [11.3]	51.4 [10.2]

Data are presented as mean [standard deviation].

*CP* chronic prostatitis/chronic pelvic pain syndrome, *DCP* diagnosed CP, *CPS* CP-like symptoms in men who have no CP diagnosis, *BPH* benign prostatic hyperplasia, *SF-12* Short-Form 12-Item Health Survey.

## Discussion

These results show that many cases of CP are not diagnosed. CPS was more than twice as prevalent as DCP (3.7% vs 1.4%). Furthermore, the QOL of men with CP was much lower than the national norm in all domains. QOL was particularly low in the RP, SF, RE, and MH domains.

Nickel et al. reported a prevalence of “chronic prostatitis like symptoms” of 9.7% from a survey done in Canada (*n* = 868) using the NIH-CPSI [5]. Subsequently, in several small studies done in limited geographical areas, the prevalence of CP has been estimated to be between 2.7% and 5.9% [6–8]. Liang et al. reported a prevalence of 8.7% for “prostatitis-like symptoms” in 12,743 people in five regions of China [9]. The sample in that study was large, but the sampling method is unclear and all of the participants were under 60 years of age. Suskind et al. estimated that the prevalence of “chronic prostatitis/chronic pelvic pain syndrome” was 1.8% (95% CI 0.9–2.7), based on data from 149 respondents after eligibility screening of 6072 randomly sampled families from across the USA [10]. The participants in our study were representative of men in Japan, given that we used quota sampling by age and place of residence, a relatively large sample size (5 010), and a wide age range (20 to 84 years). The prevalence of 5.1% (sum of DCP and CPS) in the present study is within the range reported in almost all previous studies (1.8% to 9.7%). We note that references 5 through 10 cited in this paragraph all use Nickel’s definition [5], which is likely to cover both DCP and CPS (i.e., they did not distinguish between DCP and CPS). Roberts et al. found a prevalence of DCP of 1.7% by self-report in 1543 randomly sampled residents of Olmsted County, Minnesota, USA (ages 47–90 years) [11]. That result is similar to the result of our study, although the men studied by Roberts et al. were older. Liang et al. also reported a prevalence of DCP of 4.5% [9]. Their report suggests that undiagnosed CP exists, but it was not their focus. Also, the participants in Liang’s study ranged in age from 15 to 60, while those in the present study ranged in age from 20 to 84.

The QOL of participants with CP, whether that CP had been diagnosed or was only suspected, was lower than that of the adult population of Japan as a whole. Also, on all eight domains of QOL except GH, the participants with CP had lower QOL than did those with BPH. It is noteworthy that with regard to role functioning and social functioning (i.e., RP, SF, and RE), the impact of CP on QOL was as strong as the impact of dialysis, and the impact of CP on MH scores was even stronger than the impact of dialysis [26]. These results show that men with CP have impaired physical and mental health, and that they have difficulty in their daily activities including work and socializing.

CP and depression have been reported to coexist [27]. In the present study, CP-like symptoms were associated with depression, and the prevalence of depression and of depressive symptoms was as high in the CPS group as in the DCP group (Table 1). Since this was a cross-sectional study, we could not determine whether CP caused depression, depression caused CP, or the two were independently caused by some other factor. However, it has been suggested that anxiety-like behavior may increase with CP [28], and early diagnosis and treatment of CP may help prevent depression. Participants with DCP had more comorbid conditions than did those who had CPS and those who had no CP (Table 1). This is partly attributable to age, because having DCP, having a chronic comorbid condition, being a patient, and receiving any diagnosis, are all more likely in older people. We also note that (1) participants in the CPS and the no-CP groups were similar with regard to comorbid conditions, but QOL was substantially lower in the CPS group, and (2) QOL was quite low in both the DCP and CPS groups, even though comorbidities were more common in the DCP group. Therefore, independent of comorbidities, the presence of CP itself is associated with a poor QOL.

Considering that more than 5% of the total adult male population may have CP, and that its impact on QOL appears to be substantial, CP imposes a serious health burden at the societal level. In relatively young, working-age men, CP’s largest effect on QOL is to impair their mental health and their role functioning. That is, CP lowers their productivity, which is to say that in addition to individual effects on individual patients CP also has a substantial economic impact on society [29]. This emphasizes the need to focus not only on patients in whom CP has already been diagnosed but also on men in whom CP is suspected, and particularly on younger men.

More than half of the participants with CPS were under 60 years old, and the largest gap between the prevalence of DCP and that of CPS was among men in their 30 s (Fig. 1). That is, CP seems to be seriously underdiagnosed in young men. There are several possible reasons for this finding: One is that these younger men may simply be too busy with full-time work to seek medical attention. As expected from the age distributions, full-time employment was more common among participants with CPS than among those with DCP (Table 1). Another possible explanation is that CP is not well known, especially among young people, so they may be less likely to know that they might benefit from medical attention. Furthermore, even those who do seek medical attention may not receive the correct diagnosis if they go to a clinic that does not specialize in urology [30]. Even consulting a urologist might not be enough. Liu et al. stated that urologists themselves may be confused and frustrated when treating patients who have CP. Their difficulties interpreting their patients’ complaints can lead to a lack of confidence in their diagnoses and treatments [31]. To overcome this situation, it is necessary to develop screening tools for CP in the general population, and then to provide referrals for consultation to men who test positive and thus are likely to have undiagnosed CP. Additionally, we believe that there is an urgent need to develop better tools to assist in diagnosing CP in primary care and in urology clinics. Such tools might incorporate quantitative prediction rules that use information both from patients and from physicians. This study has several limitations. First, the sample was not drawn at random. However, even with random sampling, the influence of non-respondents cannot be excluded, as the participation rate in previous studies was about 30%. We believe that the method employed in this study to allocate the number of respondents by age group and by geographic information resulted in a sample that is reasonably representative. Second, there may be misclassification of DCP because this study uses only self-reported information from the participants. It is possible that patients with a diagnosis of a similar disease, such as acute prostatitis, answered that they had received a CP diagnosis. Furthermore, since this study is based on a questionnaire survey, we cannot be sure of the accuracy of the CP diagnosis at each clinic. Third, there may also be misclassification due to the use of Nickel’s criteria for CPS. It is possible that those assigned to the CPS group were in fact affected by other diseases with similar symptoms. Specifically, some participants were at a relatively high risk for BPH because of their age, and thus the CPS group may have included men with BPH as well as men with undiagnosed CP. However, Nickel’s criteria provide the only tool that can be used in epidemiological studies, and they have been used in several previous studies. Also, the results of the two sensitivity analyses indicate that assignment to the CPS group in this study was robust to the use of definitions of CPS other than Nickel’s criteria.

## Conclusions

CP is not rare, and it has a strong negative impact on QOL. A simple, improved tool to assist in diagnosing CP should be developed. These results also provide strong evidence of the need to continue developing treatments for CP.

## Supplementary information

Appendix

Supplementary Table 1

## Data Availability

The datasets created and analyzed in this study are not publicly available. Requests for data can be addressed to the corresponding author (MS).
