# Supplementary material for: Low quality of life in men with chronic prostatitis-like symptoms
Source: Prostate Cancer Prostatic Dis. 2022 Jun 25;25(4):785–90. doi: 10.1038/s41391-022-00559-w (PMC9705241; doi:10.1038/s41391-022-00559-w)
Supplement: Supplementary file 1 — Appendix [file 41391_2022_559_MOESM1_ESM.docx]

**Appendix**

Listed below are the 12 symptoms of Chronic prostatitis/chronic pelvic pain syndrome (CP) agreed on by the panel comprising five urologists, one neurosurgeon, and two epidemiologists, with reference to the RAND/UCLA Appropriateness Method.^1^

For the two sensitivity analyses only, membership in the CPS group (the group of men who had CP-like symptoms but had not received a diagnosis of CP) was restricted to participants who had none of the following 12 symptoms. The difference between the two sensitivity analyses is explained in the main text.

Question 1: Do you have pain or discomfort in any of the following areas?

1. Area between rectum and testicles (perineum)

2. Testicles

3. Tip of penis (not related to urination)

4. Glans

5. Below the waist, in pubic or bladder area

6. Groin area

Question 2: Do you have any of the following symptoms?

1. Pain or burning during urination

2. Pain or discomfort during or after sexual climax (ejaculation)

3. Hematogenous semen or haematuria

4. Discoloration of semen

5. Premature ejaculation

6. Erectile dysfunction

**Reference:**

1. Brook RH, Chassin MR, Fink A, Solomon DH, Kosecoff J, Park RE. A method for the detailed assessment of the appropriateness of medical technologies. *Int J Technol Assess Health Care* **2**, 53-63 (1986)
